# Disseminated intravascular coagulation score predicts mortality in critically ill patients with liver cirrhosis

**DOI:** 10.1186/cc14457

**Published:** 2015-03-16

**Authors:** A Drolz, T Horvatits, K Rutter, K Roedl, S Kluge, V Fuhrmann

**Affiliations:** 1 University Medical Center Hamburg-Eppendorf, Hamburg, Germany

## Introduction

The disseminated intravascular coagulation (DIC) score is a predictor of outcome in critically ill patients [1,2]. Yet disturbances of coagulation and hemostasis, as reflected by the DIC score, are a common finding in patients with liver cirrhosis. Thus, it is unclear whether the DIC score has prognostic value in critically ill patients with liver cirrhosis. The aim of this study was to assess the applicability and prognostic impact of the DIC score in critically ill patients with liver cirrhosis.

## Methods

Patients with liver cirrhosis admitted to the medical ICU were analyzed for this study. Detailed laboratory analyses including platelet count, D-dimer, fibrinogen and prothrombin index were performed on admission and the DIC score was calculated. Survival was assessed on site or by contacting the patients or the attending physician.

## Results

In total, 150 admissions to the ICU with liver cirrhosis were analyzed. Thirty-nine percent were female. Median age was 56 (IQR 49 to 63) years. The median SOFA score on admission was 9 (6 to 13), median MELD score 26 (IQR 18 to 36). Twenty-eight-day mortality was 59%. Median DIC score on admission was 5 (IQR 4 to 6). Overt DIC (DIC score ≥5) was found in 65%. DIC score was significantly higher in nonsurvivors compared with survivors (5 (IQR 4 to 7) vs. 4 (IQR 3 to 6); *P *< 0.01). AUROC for the DIC score in prediction of 28-day mortality was 0.68 (95% CI = 0.59 to 0.77). Overt DIC on admission was significantly associated with 28-day mortality (OR = 3.4 (95% CI = 1.69 to 6.84), *P *< 0.01). The 28-day mortality rate in admissions with cirrhosis and overt DIC was 70% compared with 40% in those with a DIC score <5.

## Conclusion

Disturbances in coagulation and hemostasis are found in the majority of cirrhotic patients admitted to the ICU. The DIC score is a suitable predictor of 28-day mortality in critically ill patients with liver cirrhosis.
